# Risk factors for templating mismatch of uncemented stems in bipolar hemiarthroplasty for femoral neck fracture

**DOI:** 10.1038/s41598-023-48538-y

**Published:** 2023-11-30

**Authors:** Han Soul Kim, Sung Ha Cho, Dou Hyun Moon, Chul-Ho Kim

**Affiliations:** 1 Department of Orthopedic Surgery, Gachon University Gil Medical Center, Namdong-Gu, Incheon, Republic of Korea; 2 Department of Orthopedic Surgery, Asan Medical Center, University of Ulsan College of Medicine, 88 Olympic-Ro 43-Gil, Songpa-Gu, Seoul, Republic of Korea

**Keywords:** Risk factors, Fracture repair, Orthopaedics

## Abstract

Preoperative templating needs to be precise to optimize hip arthroplasty outcomes. Unexpected implant mismatches can occur despite meticulous planning. We investigated the risk factors for oversized and undersized stem mismatch during uncemented hemiarthroplasty using a double-tapered wedge rectangular stem for femoral neck fracture. Out of 154 consecutive patients who underwent hemiarthroplasty for femoral neck fracture, 104 patients were divided into three groups: (1) oversized (n = 17; 16.3%), (2) matched (n = 80; 76.9%), and (3) undersized stem group (n = 7; 6.7%). A smaller femoral head offset (odds ratio [OR] = 0.89, 95% confidence interval [95% CI] = 0.81–0.98, *P* = 0.017), smaller isthmus diameter (OR = 0.57, 95% CI = 0.35–0.92, *P* = 0.021), and smaller canal flare index (OR = 0.20, 95% CI = 0.04–0.98, *P* = 0.047) were significantly associated with oversized stem insertion, while older age (OR = 1.18, 95% CI = 1.01–1.39, *P* = 0.037) was associated with undersized stem insertion in logistic regression. In conclusion, when performing hemiarthroplasty for a femoral neck fracture with a double-tapered wedge rectangular stem, surgeons must pay close attention to proximal femoral geometry and patient age during preoperative planning to avoid stem mismatch.

## Introduction

The frequency of hip arthroplasty procedures has expanded in recent years due to the increase in life expectancy^1^. The goals of hip arthroplasty include relieving pain, restoring function, and correcting limb length or offset discrepancies for patient satisfaction^2^. While cemented hemiarthroplasty in older adult patients has the benefits of immediate weight bearing and a lower incidence of periprosthetic fractures, uncemented stems are associated with lower mortality rates related to cardiopulmonary complications, reduced operation time, and less blood loss^3,4^. Therefore, uncemented stems are widely used in North America and South Korea^5,6^. However, to minimize the mechanical complications of uncemented hemiarthroplasty, selecting the correct implant size is essential, as the insertion of an excessively small or large component may cause subsidence or fracture, respectively^7–9^. Therefore, the surgeon needs fastidious preoperative templating to anticipate intraoperative difficulties and thereby reduce the operation time and the risk of complications^10^.

Currently, two broad approaches for templating hip arthroplasty exist (digital vs. acetate). Both methods have undergone rigorous validation, demonstrating similar reliability and accuracy^10,11^. However, software for digital templating is expensive and requires license renewal and installation at multiple workstations in hospitals, which results in higher expenditures. In contrast, acetate templating uses acetate templates provided by the manufacturer and is more cost-effective and less time-consuming^11,12^. Therefore, many countries still rely on the acetate templating method.

Despite the importance of preoperative templating for hip arthroplasty, few studies have attempted to elucidate factors that affect the accuracy of predicting component size^10,13,14^. The following risk factors contributing to discrepancies between the preoperative template and the actual femoral implant size have been published: the planner’s lack of experience, inadequate patient positioning or mispositioning of radio-opaque reference objects in preoperative radiographs, and geometric deformity of the proximal femur^10,13–16^. However, the implant size mismatch often occurs despite proper templating by attending to adequate preoperative radiographs and patient positioning. Such unexpected implant mismatches can lead to intraoperative errors and prolong operation time. Therefore, preoperative assessment of potential risk factors for mismatch may facilitate safer operations. To date, no published articles discuss the specific implications of femoral component templating size discrepancies by referring to oversized and undersized stem mismatching, and only the technical factors mentioned above have been analyzed as risk factors.

This study aimed to investigate the accuracy of on-lay acetate templating and risk factors, especially proximal femoral morphological factors, that predict oversized or undersized stem mismatch during hemiarthroplasty for femoral neck fracture.

## Methods

### Patient selection

The study protocol was approved by the Gachon University Gil Medical Center Institutional Review Board (IRB No.: GDIRB2021-385), and the requirement for written informed consent was waived. The study was carried out by adhering to the pertinent guidelines of our institution. We reviewed consecutive patients who underwent BPHA using a single model of uncemented stem for FNF between January 2017 and February 2021 at a single institution.

**Figure 1:**
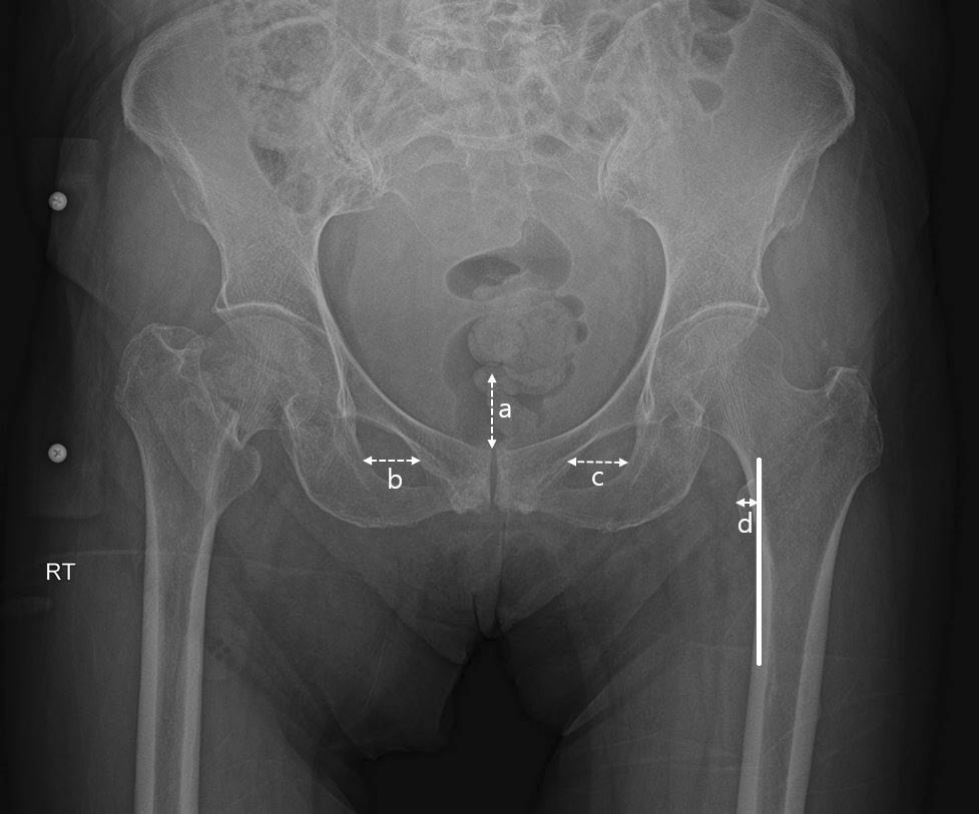
Quality assessment of a standard preoperative anteroposterior pelvic radiograph with femurs internally rotated by 15°. (**a**) Pelvic tilt was assessed by the distance from the pubis symphysis to the tip of the coccyx. (**b**) and (**c**) Pelvic rotation was assessed by first measuring the horizontal diameters of both obturator foramina and then calculating the ratio of the two measurements. (**d**) Femur rotation was evaluated by measuring the lesser trochanter thickness.

**Figure 2:**
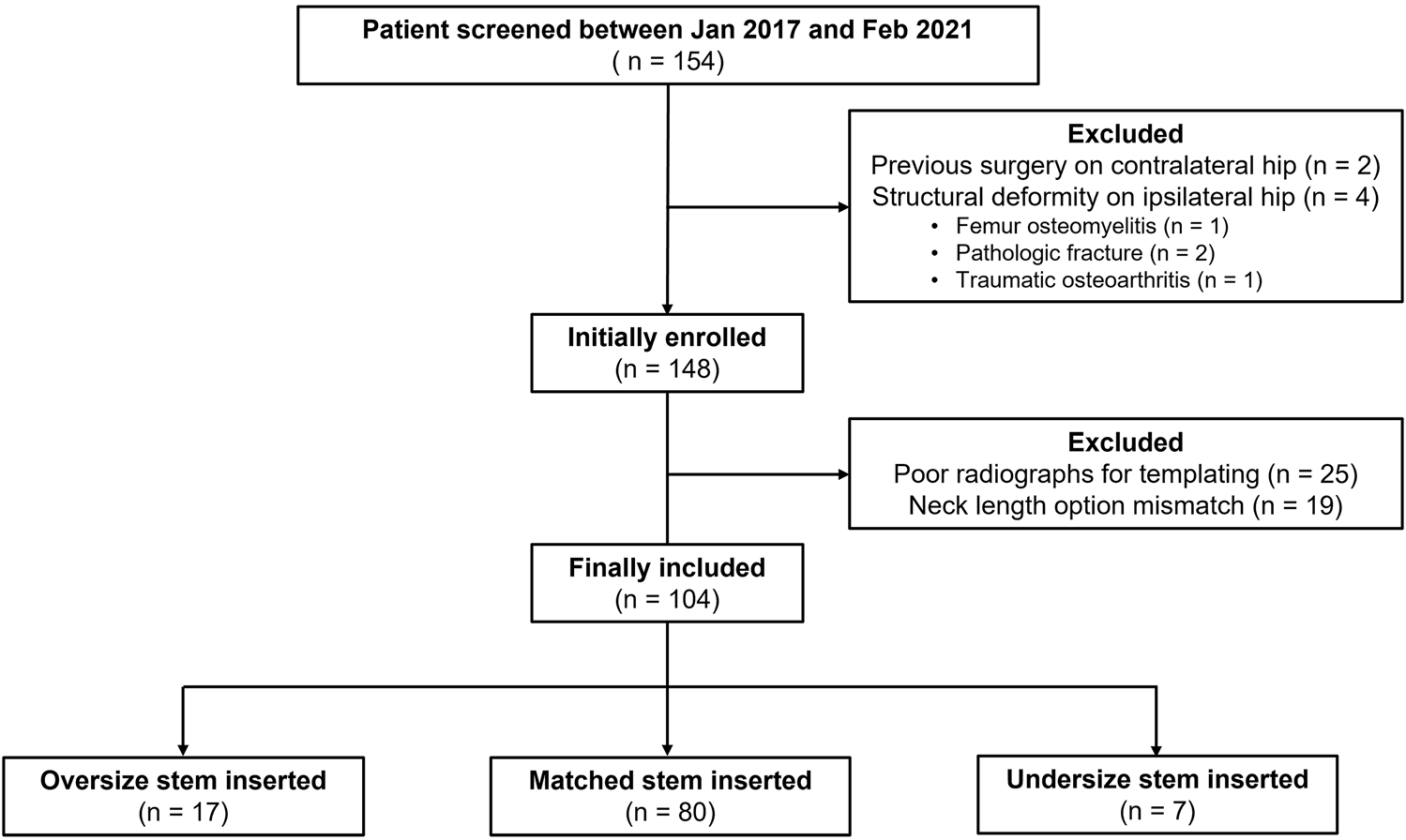
Details of the patient selection process.

The exclusion criteria were as follows: (1) previous operations on the contralateral hip (n = 2); (2) structural deformity of the ipsilateral hip (n = 4); (3) poor quality of preoperative anteroposterior (AP) radiographs (n = 25); and (4) mismatched head–neck lengths (n = 19). A quality assessment of the X-ray was performed by numerically scoring pelvic tilt, pelvic rotation, and femur rotation. Pelvic tilt was assessed by measuring the distance from the symphysis pubis to the tip of the coccyx (Fig. 1)^17^. Pelvic rotation was assessed by first measuring the horizontal distance of both obturator foramina and then calculating the ratio of the two measurements^18^. Femur rotation was evaluated by measuring the lesser trochanter thickness^19^. The measurements from each category were numerically scored, and X-ray quality was rated by the sum of these scores (Table 1). A total of 104 patients remained in the study (Fig. 2).

**Table 1:** Standard pelvic anteroposterior radiograph quality assessment scoring.

Pelvic rotation (1)	1 : rotation index = 0.7 ~ 1.5 0 : rotation index < 0.7 or > 1.5
Pelvic tilt (2)	1 : distance from pubis symphysis to coccyx tip = 1 ~ 3 cm 0 : distance from pubis symphysis to coccyx tip < 1 or > 3
Femur rotation (3)	2 : lesser trochanter thickness < 5 mm 1 : lesser trochanter thickness = 5 ~ 10 mm 0 : lesser trochanter thickness > 10 mm
X-ray quality	(1) + (2) + (3) 4 : good 3: acceptable 1–2 : poor

### Preoperative templating

We used the preoperative on-screen acetate templating method^15^. A standard preoperative AP radiograph of the hip was obtained with both femurs internally rotated by 15°. For calibration, trained radiology technicians placed a magnification marker, consisting of two round bars embedded 100 mm apart, next to the femur at the level of greater trochanter^16^. Subsequently, the image was enlarged using the zoom function of PACS PiView Star (ver. 5.0, INFINITT, Seoul, Republic of Korea) until the size of the magnification marker measured its true size of 100 mm using the magnified ruled line scale on the acetate templates.

All the preoperative templating was performed by a single hip arthroplasty specialist. To avoid measurement bias, a fellowship-trained orthopedic surgeon, who did not participate in any of the procedures included in this study, performed on-screen templating in the same manner. The two sets of template sizes were then compared using Cohen’s kappa (κ) test for interobserver agreement. Both the first and second measurements were blinded to the actual implant and index measurements. The agreement between the two investigators on on-screen templating was almost perfect (κ = 0.899), and any disagreements were resolved through discussion between the investigators.

### Surgical details

All operations were performed by a single orthopedic hip surgeon with over 30 years of experience. With the patient in the semi-lateral position, the BPHA procedure was performed using a transgluteal approach through a curvilinear incision via anterior capsulotomy. All patients were implanted with the cementless Bencox II® stem (Corentec, Seoul, Republic of Korea), a ceramic delta head, and a metal shell bipolar cup with a cross-linked polyethylene liner. The limb length discrepancy (LLD) was measured intraoperatively by palpating the tips of both medial malleoli and by confirming the C-arm images.

### Definitions of matched and unmatched stems

A matched stem was defined when the difference between a given template and the final stem component was within 1 size. When the difference was at least 2 sizes, we considered this unmatched^13^. Among patients with unmatched stems, those with a real stem size at least 2 sizes greater than the preoperative template were categorized as the oversized stem group. Patients with a real stem size at least 2 sizes smaller than the preoperative template were classified as the undersized stem group.

### Data collection

**Figure 3:**
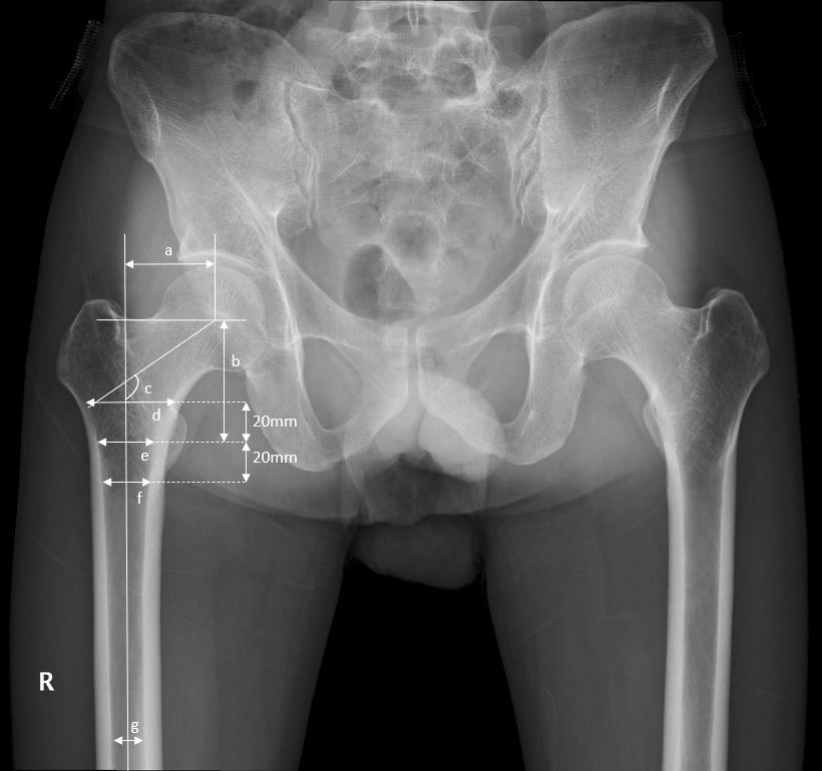
Radiologic variables of the proximal femur: (**a**) femoral head offset, (**b**) height of femoral head, (**c**) neck–shaft angle, (**d**) cavity width 20 mm above the mid-lesser trochanteric line, (**e**) cavity width at the mid-lesser trochanteric line, (**f**) cavity width 20 mm below the mid-lesser trochanteric line, (**g**) isthmus diameter.

The collected demographic data included sex, patient age, height, weight, and body mass index (BMI). We investigated the modified Koval score (MKS) for clinical variable^20^. Both bone mineral density at the femoral neck and total hip (BMD-FN and BMD-TH, respectively), which are strong indicators of osteoporosis and predictors of hip fracture, were also investigated^21,22^. The following radiologic variables were measured: Garden fracture type, Dorr type, neck–shaft angle (NSA), femoral head offset (FHO), femoral head height (FHH), cavity width 20 mm above the mid-lesser trochanter line (T + 20), cavity width at the mid-lesser trochanter line (T + 0), cavity width 20 mm below the mid-lesser trochanter line (T–20), isthmus diameter, canal flare index (CFI), and postoperative LLD (Fig. 3)^23–25^.

### Statistical analysis

For descriptive data analysis, the Shapiro–Wilk test was first performed to test normality, and data were expressed in the form of means ± standard deviation or median (range) as appropriate. Categorical variables were analyzed in terms of frequencies and proportions. Analysis of Variance (ANOVA) or Kruskal–Wallis test for continuous variables and Chi-squared or Fisher’s exact test for categorical variables were performed as appropriate for comparison among matched, undersized, and oversized stem groups. The association between each variable and the templating mismatch was analyzed using logistic regression. Variables of interest were reexamined via multivariable models using the backward elimination method, and *P* < 0.05 was considered statistically significant. Data analyses were conducted using PASW Statistics for Windows, version 18.0 (SPSS Inc., Chicago, IL, USA).

## Results

Eighty patients (76.9%) were assigned to the matched stem group (size discrepancies ≤ 1), 17 (16.3%) were assigned to the oversized stem group (with mismatches ranging from 2–3 sizes), and seven (6.7%) were assigned to the undersized stem group (with mismatches ranging from 2–4 sizes). Seventy-two patients (69.2%) were female, and the mean age and BMI were 78.7 years (range, 58–93 years) and 22.1 kg/m^2^ (range, 15.0–30.2 kg/m^2^), respectively. Most of the patients were household ambulators with MKS of ≥ 3 (Grade 3: n = 31, 29.8%; Grade 4: n = 41, 39.4%; Grade 5: n = 22, 21.2%). The mean T-scores of BMD-FN and BMD-TH were − 2.4 ± 1.0 and − 2.7 ± 1.2, respectively. In terms of radiographic profiles, Garden type IV (n = 68, 65.4%) and Dorr type B (n = 69, 66.3%) were the most common. The mean NSA, FHO, and FHH were 131.0° (range, 118.8–166.1°), 40.6 mm (range, 16.7–63.3 mm), and 72.2 mm (range, 58.8–90.8 mm), respectively. The mean T + 20, T + 0, and T-20 were 42.9 mm (range, 25.3–60.9 mm), 29.6 mm (range, 18.7–46.8 mm), and 22.0 mm (range, 12.8–47.3 mm), respectively. The mean isthmus diameter and CFI were 13.8 mm (range, 8.6–20.4 mm) and 3.2 mm(range, 1.7–4.7 mm), respectively. The mean postoperative LLD was 0.9 mm (range, 0–3.7 mm). More details about patient demographics, including overall and subgroup data, are presented in Table 2.

**Table 2:** Demographic, clinical and radiologic parameters for the patient group with an oversized, matched, and undersized stem insertion during bipolar hemiarthroplasty using type 3C rectangular stem.

Variables	Total (n = 104)	Oversized (n = 17)	Matched (n = 80)	Undersized (n = 7)
Male sex	72 (69.2%)	11 (64.7%)	55 (68.7%)	6 (85.7%)
Age (yrs)	78.7 ± 7.9 (58–93)	76.5 ± 8.0 (63–90)	78.6 ± 7.8 (58–93)	84.7 ± 7.3 (71–90)
BMI (kg/m^2^)	22.1 ± 3.2 (15.0–30.2)	22.7 ± 3.7 (17.5–30.2)	22.1 ± 3.2 (15.0–28.6)	21.0 ± 1.4 (19.6–22.8)
BMD-hip	− 2.4 ± 1.0 (− 4.6– − 1.1)	− 2.1 ± 1.3 (− 4.0– − 1.1)	− 2.4 ± 0.9 (− 4.6– − 0.7)	− 2.8 ± 1.0 (− 4.0– − 1.1)
BMD-total	− 2.7 ± 1.2 (− 5.8–2.3)	− 2.5 ± 1.4 (− 4.8–0.6)	− 2.7 ± 1.2 (− 5.8–2.3)	− 3.2 ± 1.0 (− 4.5– − 1.8)
mKoval (1:2:3:4:5:6)	22 (21.2%): 41 (39.4%): 31 (29.8%): 8 (7.7%): 2 (1.9%): 0 (0%)	5 (29.4%): 5 (29.4%): 5 (29.4%): 2 (11.8%): 0 (0%): 0 (0%)	16 (20.0%): 34 (42.5%): 22 (27.5%): 6 (7.5%): 2 (2.5%): 0 (0%)	1 (14.3%): 2 (28.6%): 4 (57.1%): 0 (0%): 0 (0%): 0 (0%)
Garden type (I: II: III: IV)	5 (4.8%): 4 (3.8%): 27 (26%): 68 (65.4%)	1 (5.9%): 0 (0%): 9 (52.9%): 7 (41.2%)	4 (5%): 4 (5%): 14 (17.5%): 58 (72.5%)	0 (0%): 0 (0%): 4 (57.1%): 3 (42.9%)
Dorr type A:B: C	30 (28.8%): 69 (66.3%): 5 (4.8%)	5 (29.4%): 12 (70.6%): 0 (0%)	25 (31.3%): 52 (65%): 3 (3.8%)	0 (0%): 5 (71.4%): 2 (28.6%)
NSA (°)	131.0 ± 6.6 (118.8–166.1)	131.4 ± 6.4 (118.8–144.6)	131.0 ± 6.5 (120.5–166.1)	130.2 ± 9.5 (119.7–144.2)
FHO (mm)	40.6 ± 7.3 (16.7–63.3)	36.8 ± 8.4 (16.7–51.2)	41.1 ± 6.6 (27.7–56.6)	44.2 ± 9.8 (34.3–63.3)
FHH (mm)	72.2 ± 7.1 (58.8–90.8)	71.1 ± 6.7 (61.2–84.0)	72.3 ± 7.2 (58.8–90.8)	74.1 ± 8.1 (60.4–82.0)
T + 20 (mm)	42.9 ± 7.6 (25.3–60.9)	39.8 ± 6.6 (30.2–51.9)	43.0 ± 7.5 (25.3–58.9)	49.6 ± 7.4 (40.6–60.9)
T + 0 (mm)	29.6 ± 5.5 (18.7–46.8)	28.3 ± 6.1 (22.8–46.8)	29.5 ± 5.3 (18.7–41.2)	33.9 ± 4.3 (28.0–39.7)
T − 20 (mm)	22.0 ± 4.8 (12.8–47.3)	19.8 ± 4.0 (13.5–31.8)	22.1 ± 4.9 (12.8–47.3)	25.7 ± 3.5 (22.0–30.3)
Isthmus (mm)	13.8 ± 2.3 (8.6–20.4)	12.9 ± 1.7 (10.1–15.9)	13.8 ± 2.3 (8.6–20.4)	15.8 ± 2.1 (12.4–18.8)
CFI	3.2 ± 0.6 (1.7–4.7)	3.1 ± 0.5 (2.1–4.0)	3.2 ± 0.6 (1.7–4.7)	3.2 ± 0.5 (2.5–4.0)
LLD (mm)	0.9 ± 0.9 (0–3.7)	1.1 ± 1.1 (0–2.8)	0.9 ± 0.9 (0–3.7)	0.9 ± 0.6 (0–1.6)

*BMI* body mass index; *BMD-hip* bone mineral density in the hip; *BMD-total* bone mineral density in total; *mKoval* modified Koval grade; *NSA* neck-shaft angle; *FHO* femoral head offset; *FHH* femoral head height; *T* + *20* Cavity width 20 mm above the mid-lesser trochanter line; *T* + *0* cavity width at the mid-lesser trochanter line; *T-20* cavity width 20 mm below the mid-lesser trochanter line; *CFI* canal flare index; *LLD* limb length discrepancy.

### Risk factor analysis for templating mismatch

The univariate and multivariable logistic regression templating mismatch risk factor analysis results are presented in Tables 3 and 4. A smaller FHO (odds ratio [OR], 0.89; 95% confidence interval (CI), 0.81–0.98; *P* = 0.017), a smaller isthmus diameter (OR, 0.57; 95% CI, 0.35–0.92; *P* = 0.021), and a smaller CFI (OR, 0.20; 95% CI, 0.04–0.98; *P* = 0.047) were significantly associated with oversized stem insertion in the multivariable logistic regression model (Table 3). Conversely, older patient age (OR, 1.18; 95% CI, 1.01–1.39; *P* = 0.037) was the only factor that was significantly associated with undersized stem insertion in the multivariable logistic regression model (Table 4).

**Table 3:** Results of univariate and multivariable logistic regression analysis for potential risk factors associated with oversized stem insertion.

Characteristic	Univariate analyses		Multivariable analyses	
	OR (95% CI)	*P* value	OR (95% CI)	*P* value
Sex (Male)	0.52 (0.04–6.41)	0.610	–	–
Age (yrs)	0.94 (0.84–1.05)	0.282	–	–
BMI (kg/m^2^)	2.00 (0.13–30.10)	0.617	–	–
BMD-hip	3.41 (0.92–12.68)	0.067	1.71 (0.94–3.12)	0.079
BMD-total	0.58 (0.18–1.93)	0.374	–	–
NSA (°)	0.89 (0.77–1.03)	0.107	–	–
FHO (mm)	0.83 (0.72–0.95)	0.007	0.89 (0.81–0.98)	0.017
FHH (mm)	0.93 (0.82–1.05)	0.225	–	–
T + 20 (mm)	1.70 (0.76–3.80)	0.199	–	–
T + 0 (mm)	1.23 (0.98–1.55)	0.074	1.15 (0.98–1.34)	0.089
T-20 (mm)	0.79 (0.57–1.10)	0.164	–	–
Isthmus (mm)	0.11 (0.01–1.30)	0.080	0.57 (0.35–0.92)	0.021
CFI	0.00 (0.00–4.98)	0.100	0.20 (0.04–0.98)	0.047

*BMI* body mass index; *BMD-hip* bone mineral density in the hip; *BMD-total* bone mineral density in total; *NSA* neck-shaft angle; *FHO* femoral head offset; *FHH* femoral head height; *T* + *20* Cavity width 20 mm above the mid-lesser trochanter line; *T* + *0* cavity width at the mid-lesser trochanter line; *T-20* cavity width 20 mm below the mid-lesser trochanter line; *CFI* canal flare index.

**Table 4:** Results of univariate and multivariable logistic regression analysis for potential risk factors associated with undersized stem insertion.

Characteristic	Univariate analyses		Multivariable analyses	
	OR (95% CI)	*P* value	OR (95% CI)	*P* value
Sex (Female)	30.63 (0.02–52,632.60)	0.368	–	–
Age (yrs)	1.34 (0.87–2.08)	0.188	1.18 (1.01–1.39)	0.037
BMI (kg/m^2^)	1.32 (0.001–1436.59)	0.938	–	–
BMD-hip	1.12 (0.12–10.52)	0.924	–	–
BMD-total	2.36 (0.51–10.88)	0.271	–	–
NSA (°)	1.13 (0.87–1.47)	0.377	–	–
FHO (mm)	1.03 (0.83–1.29)	0.787	–	–
FHH (mm)	1.22 (0.90–1.64)	0.203	–	–
T + 20 (mm)	0.82 (0.34–1.98)	0.658	–	–
T + 0 (mm)	1.63 (0.73–3.63)	0.233	1.22 (0.99–1.51)	0.067
T-20 (mm)	0.93 (0.54–1.60)	0.796	–	–
Isthmus (mm)	2.98 (0.18–48.31)	0.442	1.49 (0.92–2.41)	0.105
CFI	13.59 (0.00–2,265,235.04)	0.671	–	–

*BMI* body mass index; *BMD-hip* bone mineral density in the hip; *BMD-total* bone mineral density in total; *NSA* neck-shaft angle; *FHO* femoral head offset; *FHH* femoral head height; *T* + *20* Cavity width 20 mm above the mid-lesser trochanter line; *T* + *0* cavity width at the mid-lesser trochanter line; *T-20* cavity width 20 mm below the mid-lesser trochanter line; *CFI* canal flare index.

## Discussion

Preoperative templating needs to be precise to optimize hip arthroplasty outcomes. However, unexpected implant mismatches can occur despite meticulous planning, which may lead to intraoperative errors and prolonged operation time. Therefore, preoperative assessment of potential risk factors for mismatch may aid the surgeons in anticipating “the unexpected” and facilitating safer operations. To date, no published articles discuss the specific implications of femoral component templating size discrepancies by referring to oversized and undersized stem mismatching. Existing analyses have predominantly focused on technical factors, such as inadequate radiographs due to mispositioned patients and markers. We investigated the risk factors for oversized and undersized stem mismatch during uncemented hemiarthroplasty using a double-tapered wedge rectangular stem for femoral neck fracture. The main findings of our study were that the risk factors for oversized stem insertion in hip arthroplasty were small FHO, isthmus diameter, and CFI, and that older patient age was a risk factor for undersized stem insertion.

Few previous studies have investigated risk factors associated with discrepancies between preoperative templates and the actual femoral implant size^10,13,14^. Identified risk factors for templating errors encompass obesity, male sex, and the planner’s level of experience. However, these studies did not specifically investigate how these risk factors could affect templating errors. Instead, they assumed technical errors, such as poor radiograph quality, mispositioning of patients or radio-opaque reference objects, and quality of radiographs, to be the main factors associated with templating mismatches. Moreover, to our knowledge, no published studies have evaluated the risk factors for size differences between preoperative templates and inserted stems by categorizing oversized and undersized stem insertions separately.

Our findings are intriguing since a smaller FHO, isthmus diameter, and CFI all seem to be favorable for smaller-size stem insertion. An explanation for these findings might be that we paid close attention to the distal part of femoral geometry when performing preoperative templating, thereby leading to underestimations of overall stem sizes, resulting in templating mismatches. Additionally, since this study utilized type 3C stems, characterized by distal narrowing in both the AP and lateral–medial aspects, stem fitting was primarily influenced by the proximal width than the distal width^26^. In other words, the relatively wider proximal diameter of the femur could have more influence on stem size than the relatively narrow distal diameter of the femur. Further research will be needed to evaluate the various stem designs in this regard. The second explanation for oversized stem insertion might be that a smaller isthmus diameter and CFI indicate relatively healthy and non-osteoporotic bone, which means that the femur has enough inner cortical margin for rasping, leading to a larger stem size than templated. Conversely, osteoporotic bone with a larger isthmus diameter and CFI lacks sufficient inner cortex for rasping, which results in the real stem fitting true to the templated size. Notably, significant intergroup differences in terms of BMD, MKS, or T-scores were not observed.

In terms of undersized stem insertion, older age was the sole significant risk factor. Previously, Carter et al. found sclerotic bone to be a risk factor for size discrepancies between preoperative templates and actual femoral stems^10^. Additionally, a seminal biomechanical study demonstrated age-related variation in bone elasticity in the human proximal femur^27^. Our results are concordant in that the low elasticity and stiffening associated with aging bone tissue may be conducive to undersized stem insertion. Moreover, a notably low mean BMD-TH (− 3.2 ± 1.0) may insinuate that the femoral bone quality in the undersized group was poor. Consequently, the surgeon may have either broached conservatively or chosen a smaller stem in concern for intraoperative periprosthetic fracture.

Some other variables, such as sex and BMI, have also been identified as risk factors for template mismatching in other studies. Dammerer et al. demonstrated greater accuracy of preoperative templating in females than males, especially with the femoral component in hip arthroplasty procedures^13^. Although we did not identify sex as a significant risk factor for oversized or undersized stem insertion, the oversized stem group had a higher proportion of women, and the undersized stem insertion group consisted predominantly of men (Table 2). Debate exists regarding the association between patient BMI and the risk of templating errors in hip arthroplasty. Holzer et al. found that high BMI affects femoral implant size estimations during THA^14^. They suggest that BMI is a risk factor for templating error since a high BMI could affect the position of the magnification marker in the preoperative radiographs. Contrastingly, BMI was not a risk factor for templating errors in the study by Sershon et al.^28^. Additionally, Riddick et al. concluded that no significant association exist between BMI and templating accuracy^29^. Recently, Dammerer et al. also found that patient BMI was not associated with templating accuracy in their 620 uncemented primary THA cases^13^. This aligns with our study findings, as we also did not find a significant association between BMI and templating mismatch. Considering that our study double-checked X-ray quality by first having trained radiology technicians perform examinations with a well-established protocol and then implementing secondary quality assessments to exclude radiographs with poor quality, we further strengthened the argument that BMI is not a risk factor for templating mismatch.

A strength of our study was that all of the included patients underwent BPHA. Previous studies investigating risk factors for templating precision have primarily focused on THA patients or a combination of THA and BPHA patients^10,13,14^. However, conducting a study on template precision based on THA may be difficult since femoral stem size may have to be adjusted according to intraoperative changes in the hip center position. Therefore, our study only focused on factors that affect stem sizing during BPHA and excluded cases in which head–neck lengths differed from the corresponding templates. Acetabular positioning and rotational center of the hip may depend on the surgeon’s preference; therefore, focusing only on the femoral stem in BPHA cases that are free from changes in rotational center allowed us to eliminate potential bias and isolate the true risk factors for stem templating errors, which strengthened our study findings.

Previous studies have indicated that the acetate templating method is associated with templating mismatch rates between 5 and 12%. Carter et al. found an 88–95% accuracy rate (i.e., the rate of femoral stem implantation within a ± 1-size error margin) associated with preoperative templating for uncemented THA or BPHA^10^. Unnanuntana et al. also reported a template mismatch rate of about 10% (with respect to a ± 1-size error margin) in their study of 109 cementless THAs^30^. Recently, Dammerer et al. reported a 90% stem templating accuracy rate, in which accurate templating was defined as within ± 1 size^13^. In our study, the templating accuracy within 1 size was nearly 80%. We believe that the accuracy rate was negatively affected by the small sample size. Further larger-scale multicenter research may yield higher accuracy rates.

Another argument for the lower accuracy rate in our study could be that analog templating, instead of digital templating, was used. Although several software packages and artificial intelligence algorithms are available for digital templating, analog templating has been proven to have satisfactory accuracy, easy accessibility, and excellent cost-effectiveness^15,31–33^. In comparative studies, the accuracy of analog templating of the cup and femur published in the literature ranges from 60 to 97% and 52 to 98%, respectively. The accuracy of digital templating of the cup and femur rages from 52 to 81% and 50 to 94%, respectively^11,12,16,31,33–36^. Several studies have even indicated that acetate templating is superior to digital templating^11,12,16,33–35^. Moreover, analog acetate templating and hybrid on-lay templating are still widely used due to the inaccessibility of digital templating software^15,37^. Considering the cost-effectiveness and comparable accuracy and reliability of both techniques, conducting a study using a more accessible and cost-effective templating method can be considered a strength of our study, as it increases its generalizability.

Our study had some limitations. We only investigated type 3C stems. Although this may be a strength in that the results have unity and consistency, it would be useful to include the risk factors associated with other widely used stems, such as type 1 stems and proximal-coated tapered wedge stems. Additionally, we did not investigate the long-term clinical follow-up results of the templating mismatch cases. Further long-term research is necessary to clarify the long-term significance of templating mismatches. Regardless, to our knowledge, this is the first study that attempts to elucidate potential risk factors for template mismatch. Preoperative templating is crucial for surgeons to anticipate intraoperative difficulties, reduce operation time, and carry out safer operations. Therefore, we believe that this study has value in its attempt to search for risk factors and leads the way to future studies with various types of stems.

In conclusion, when performing hemiarthroplasty for a femoral neck fracture with a double-tapered wedge rectangular stem, surgeons must pay close attention to the proximal femoral geometry and patient age during preoperative planning for the possibility of stem mismatch and perhaps consider other options for femoral stems.

## Informed consent

The requirement for written informed consent was waived by the Gachon University Gil Medical Center Institutional Review Board, and the study was carried out by adhering to the pertinent guidelines of our institution.

## Data Availability

The datasets generated and analyzed during the current study are not publicly available since they contain potentially identifiable information for each patient; however, they are available from the corresponding author upon reasonable request.
